# Barriers and Enablers for Sustaining Nurse-Led Use of Clinical Decision Support Tools for Antibiotic Stewardship: Qualitative Study

**DOI:** 10.2196/83567

**Published:** 2026-03-10

**Authors:** Victoria L Tiase, Aurie D Tovar, Natalie Henning, Mariana Braga, Keelin McHugh, Lynn Xu, Haddy Bah, Alice Yuroff, Rachel Hess, Devin M Mann, David Feldstein, Elizabeth R Stevens

**Affiliations:** 1 Department of Biomedical Informatics, University of Utah, 421 Wakara Way, Salt Lake City, UT, 84112, United States, 1 9174707754; 2 Department of Population Health, NYU Grossman School of Medicine, New York, NY, United States; 3 Department of Medicine, University of Wisconsin School of Medicine and Public Health, Madison, WI, United States; 4 Department of Population Health Sciences, University of Utah, Salt Lake City, UT, United States

**Keywords:** clinical decision support systems, electronic health records, nursing, antibiotic stewardship, ambulatory care facilities

## Abstract

**Background:**

Clinical decision support (CDS) tools embedded in electronic health records in the form of integrated clinical prediction rules provide a potentially effective intervention to reduce inappropriate antibiotic prescribing for acute respiratory infections. However, their effectiveness has been limited by workflow barriers and low adoption by health care providers. Nurses are well positioned to implement evidence-based protocols using CDS tools. In a multicenter randomized controlled trial, a nurse-led implementation strategy for acute respiratory infection integrated clinical prediction rules was evaluated for use in primary care and urgent care settings.

**Objective:**

This study aimed to examine nurse and nurse leader perspectives on the sustainability of an electronic health record–integrated CDS tool for antibiotic stewardship and explored factors influencing its potential long-term integration into ambulatory nursing practice beyond the clinical trial.

**Methods:**

We interviewed 22 nurses and nurse leaders from 37 clinics across 3 academic medical centers that participated in the clinical trial. Two semistructured interview guides, one for nurses and one for nursing leadership, were developed to understand the barriers and facilitators to implementing a decision aid tool for nurses and to elicit challenges specific to nursing interactions with the CDS tool. Interviews were recorded and transcribed. Using thematic content analysis and iterative coding, our team collaboratively identified emerging themes related to sustainability and refined the results with consensus.

**Results:**

Five themes emerged: (1) importance of staffing stability and capacity, (2) impact of dedicated clinic resource availability, (3) variable nurse readiness with CDS-guided clinical care, (4) influence of openness to change and a nurse-supportive clinic culture, and (5) ongoing need for training and support. Specific recommendations for future actions were also noted.

**Conclusions:**

Our findings revealed specific barriers and facilitators to the sustainability of a CDS tool from the nursing perspective that can inform further implementation of nurse-led delegation protocols in the ambulatory setting. Future solutions should consider mapping physical workflows, scheduling specific to nurse visits, continuing education, and treating cough and sore throat as 2 distinct processes.

## Introduction

Antibiotic resistance is a growing global health concern, exacerbated by the overprescription of antibiotics for acute respiratory infections (ARIs) [1]. Despite available evidence-based guidelines and clinical decision support (CDS), physicians often prescribe antibiotics inappropriately due to perceived patient expectations, time pressures, and cognitive fatigue [2]. To address this issue, clinical prediction rule (CPR) systems have been developed and integrated as integrated clinical prediction rules (iCPRs) into electronic health records (EHRs) as CDS to assist physicians in making informed antibiotic prescribing decisions [3,4]. However, their impact has been limited by low provider adoption rates [5,6]. Evidence suggests that registered nurses (RNs) have the potential to implement evidence-based protocols [7,8], participate in antimicrobial stewardship [9], achieve high patient satisfaction and symptom resolution [7], and demonstrate higher adoption rates of CDS tools than physician-led initiatives [10].

Building on this potential, the multicenter, step-wedge, cluster randomized controlled trial (iCPR3 RCT) evaluated whether RNs could effectively use iCPR tools to guide evidence-based care for patients presenting with ARIs in ambulatory care settings [11,12].

An early implementation assessment identified several potential barriers and facilitators to adopting the nurse-driven CDS tools [10]. As the iCPR3 RCT concluded, we sought to understand whether nursing departments would consider continuing to use iCPR tools in clinical practice after research support was withdrawn. We also aimed to identify barriers that may hinder sustained use within ARI workflows beyond the study period. By examining these perspectives, our goal was to identify strategies to sustain nurse-led use of iCPR tools in clinical practice as a means to improve patient outcomes [13].

## Methods

### Research Design

We used semistructured interviews and thematic analysis to examine the perspectives of RNs and nurse leaders regarding the sustainability of the iCPR tools, including aspects of implementation, usability, and perceived impact. For the purposes of this study, we defined sustainability as the extent to which nurses would continue to use a tool as part of standard clinical workflows beyond the study period and without research personnel implementation support or incentives.

### Ethical Considerations

The study protocol and procedures were approved by the NYU Langone Health Institutional Review Board (NYULH study number i19-01222), which served as the study’s single institutional review board. We received a waiver of written informed consent for this study. All study data reported in this manuscript are deidentified. Compensation was provided in accordance with institutional policies.

### Nurse iCPR Intervention

The nurse intervention used a structured delegation protocol, wherein the specific clinical task of assessing the need for antibiotics in low-acuity ARI patients was transferred from a physician to an RN. Provider-to-nurse delegation is operationalized through protocol-driven authorization of nursing tasks by a licensed provider, with accountability retained by the delegating provider. In the iCPR3 RCT, the intervention consisted of triage followed by in-person CDS-guided RN visits for patients with low-acuity ARI symptoms. The EHR-integrated CDS included a triage note template to assess symptoms and acuity and 2 reason-for-visit-specific (ie, sore throat or cough) note templates with iCPRs that guide RNs to complete an evidence-based risk calculator as part of the visit [12]. The resulting risk score is linked to an order set for diagnostic testing, prescriptions, and patient instructions.

All tasks performed by RNs as part of the intervention—triage, symptom assessment, use of CDS tools, and execution of evidence-based care pathways—are well within the scope of nursing practice and align with their clinical training. The iCPRs used were previously found to be effective for reducing inappropriate antibiotic prescribing when used by physicians [2,4,11]. The intervention aimed to standardize care delivery and reduce unnecessary antibiotic use in outpatient settings.

The intervention was implemented as a pragmatic stepped-wedge cluster randomized trial across 43 primary and urgent care practices in 3 academic health systems affiliated with the University of Utah, the University of Wisconsin, and NYU Langone. To be eligible for participation, practices were required to have at least 1 RN full-time equivalent capable of performing triage within the EHR and conducting RN on-site visits. Nurses in the intervention group received 1 hour of online training prior to 1 hour of in-person training on how to use the iCPR tool, including background on the iCPRs, EHR-based walkthroughs of using the iCPR tool within the CDS, demonstration videos simulating the tool in a live clinical encounter, and clinical training for physical examinations [11]. Complete study procedures are reported elsewhere [12].

### Study Design

We conducted remote video interviews (via Zoom and Webex) lasting approximately 30 minutes between September 2024 and January 2025. Each participant was interviewed by a trained research team member from the corresponding health system (NH, KM, AY, VT) using 1 of 2 semistructured interview guides. Our team developed these interview guides, 1 for RNs and 1 for leadership, based on a series of open-ended structured questions previously used in a validated survey to understand the barriers and facilitators to implementing a lupus decision aid tool for nurses [14]. We adapted the questions to explore relevant Consolidated Framework for Implementation Research (CFIR) constructs, as well as address conditions specific to the ambulatory care environment and the iCPR intervention; we retained all other aspects of the survey [15]. Interviews consisted of questions to understand barriers and facilitators related to tool use, clinical team interactions, what worked well, any competing priorities, strategies for mitigating issues, and future adoption sustainability (see Tables S1 and S2 in Multimedia Appendix 1). Probing questions were used to verify the interpretation of participant responses, and recommendations were solicited for continued use of the iCPR tool in clinical workflows.

### Participants

We recruited a convenience sample of RNs and nurse leaders (did not have to be an RN) from primary care clinics and urgent care centers participating in iCPR3. Participants were considered eligible if they expressed familiarity with the iCPR tool and, if they were nurse participants, had completed a nurse visit using the tool. Participants were recruited via email by study personnel at each site. Recruitment procedures varied slightly by study site due to local policies and conditions. Specifically, given the proportionately large number of clinics at the University of Wisconsin (21 clinics vs 9 and 7 clinics at NYU and Utah, respectively) and with the objective of obtaining diverse perspectives, participants were recruited as a stratified convenience sample from the top and bottom 5 performing clinics, based on the average number of nurse visits completed per week per clinic volume at randomization. In addition, University of Utah participants were offered a US $25 gift card as an incentive, whereas other study sites did not provide financial incentives due to institutional policies around employee compensation. A member of the study team verbally reviewed the study information with each participant to ensure understanding; all participants then provided verbal agreement to participate.

### Thematic Analysis Procedures

We conducted a thematic content analysis to identify themes related to sustainability by extracting high-level themes from the data while examining the frequency of concepts or keywords [16]. Each interview was audio-recorded and transcribed using an automated service (Landmark Associates). One team member (NH) reviewed each file for transcription accuracy prior to analysis. Transcripts were analyzed, and a codebook was developed using Dedoose [17].

We first conducted a joint analysis of 2 interviews to allow the study team members to become accustomed to the codebook, make updates to codes, and resolve conflicts. All study team members were trained in qualitative techniques. Next, 2 team members coded each transcript independently, and then all coders (VT, NG, MB, KM, AT) met as a team to compare and resolve disagreements. During this process, we reviewed for thematic saturation, ensuring the sample met this criterion based on the diminishing need to update code definitions. Our analysis focused on the barriers and facilitators to sustainability of iCPR tool use beyond the study end date. Once all transcripts were coded, we reviewed the data code by code to ensure definitions had remained consistent. All coders then reviewed each excerpt to collectively derive emerging themes related to barriers, facilitators, and recommendations for solutions. Emerging themes were grouped into high-level categories through consensus. This study adhered to the COREQ (Consolidated Criteria for Reporting Qualitative Research) guidelines for qualitative studies involving interviews and focus groups [18].

## Results

### Descriptive Results

We invited 56 eligible individuals to participate and conducted 22 individual interviews across the 3 sites, each lasting 15 to 45 minutes. Participants included 9 nurses (1 NYU, 8 Wisconsin) and 13 nurse leaders (4 NYU, 2 Utah, 7 Wisconsin); demographic information was not collected from either group. Due to limited tool use during the study, no nursing participants from the University of Utah elected to participate in the interviews.

Through thematic analysis, we identified 5 overarching themes related to nurses’ and nurse leaders’ perspectives on the implementation, effectiveness, and sustainability of the iCPR intervention as part of standard care processes. The 5 themes that emerged included: (1) importance of staffing stability and capacity; (2) impact of dedicated clinic resource availability; (3) variable nurse readiness with CDS-guided clinical care; (4) influence of openness to change and a nurse-supportive clinic culture; and (5) ongoing need for training and support.

### Importance of Staffing Stability and Capacity

One of the most prominent themes concerned the need to have sufficient trained RNs staffed and available in the clinic to support the time needed to triage patients and conduct nurse visits. In addition to nurse staffing, participants emphasized concerns related to physician and support staff levels. Contributing to these concerns, many participants (20, 91%) discussed issues related to staff turnover, including the challenges of retaining staff, training new staff, and implications for using the iCPR tool workflows.

When questioned about barriers to using the iCPR tool, one nurse stated:

*We are staffed with only three nurses, which is not a lot… One of our nurses left in May of last year to work elsewhere. Then, we hired a new nurse. Our third nurse went on maternity leave. Then when she came back, the other nurse had to leave for other reasons too. We've kind of just been at this [struggle] of not being able to consistently have a good number of staffing.*

[N 3.18]

The shortage of nursing support staff often required nurses to cover multiple roles, making it challenging to prioritize tasks and manage workload effectively. One participant stated:

*…some clinics can’t handle it… they don’t have enough people to even answer the phones.*

[N 3.3]

With full staffing, nurses expressed confidence in executing the iCPRs:

*We are pretty much fully staffed… it has not been a burden to participate and be a part of this survey and process… it’s certainly helped our patients.*

[N 3.3]

*We could have two nurses and visits at the same time and patients aren't having to wait.*

[N 3.2]

Additionally, staff turnover was linked to challenges in familiarizing and educating new staff regarding the iCPRs due to limited training time. Increasing awareness among new nurses completing orientation who lacked exposure to the study was discussed. One nurse stated:

*Now that I'm thinking about it, there’s a whole bunch of nurses that started that they weren't probably there when it first started. They don't even know about it, and I don't know if it’s part of their orientation packet.*

[N 1.7]

A key subtheme involved virtual triage coordination, where nurses working remotely were able to triage patients by phone and monitor clinic appointment availability. Approximately one-quarter of the participants (6, 27%) felt that this workflow disrupted nurse visit appointment availability.

*It’s a small nursing team so you have anywhere from two to three nurses working on a given day and then with the nurses that have the option to work remote now sometimes you only have one RN [registered nurse] in clinic. That impacts availability for appointments, so I think that is always a challenge.*

[N 3.11]

Another participant expressed difficulties coordinating with colleagues not physically in the same place, since the phone triage nurse at Wisconsin and NYU sites was typically different from the nurse who conducts the in-person nurse visit, highlighting staffing coordination in a hybrid care setting.

*Sometimes, if you're at home triaging the patient, and they could use a nurse visit, trying to coordinate that with colleagues can be hard if it’s a busy day. You don't really know what’s going on over there [in the clinic]…*

[N 3.15]

### Impact of Dedicated Clinic Resource Availability

The impact of clinic resource availability, including physical space for nurse visits and scheduling accessibility, was identified as a barrier to uptake of the in-person nurse visit component of the iCPR workflow. The ability to manage the patient care continuum was disrupted when examination rooms were unavailable. One nurse stated:

*…we don't necessarily have a place to go with our patients*…*again, sometimes we are just running around trying to find a location for the patient to be assessed in and to complete the visit in.*

[N 3.4]

Discussion of the physical clinic setup highlighted that the lack of available examination rooms posed challenges. One RN stated:

*We've always had to kind of just find a room on the fly for any of our nurse visit types each day, whether they're iCPR or not. A day like today, all of our exam rooms are occupied by the providers that are here. Our plan is just when the patient comes, if there is an available room, just kind of quickly utilize that.*

[N 3.18]

Alternatively, when the clinic set aside dedicated rooms for nurse visits and triage, it reportedly streamlined workflows and supported iCPR tool uptake.

*Structurally, we had a room all ready to do nurse visits that we do for other things. That was easy enough to adopt this [iCPR] into that.*

[N 3.3]

*We have a designated room for nurse visits every day ’cause we have nurse visits every day, and it’s an exam room at the front of the clinic, and we have everything that we need in there.*

[N 3.10]

Coupled with dedicated room space, time set aside for nursing appointments facilitated iCPR care workflows. One clinic workaround included having patients with sore throats or coughs come in at prescheduled time slots so that nurses and testing resources (such as throat swabs) were available. This allowed the clinic to structure the schedule in a way that allowed nurses to complete patient care activities.

*I know that one of the things that we tried to focus on and try to do too, as far as our department, is we tried to make sure that we had like the 11:00 and 1:00 p.m. slots available for the nursing and cough and sore throat visits…*

[N 3.4]

### Variable Nurse Readiness With CDS-Guided Clinical Care

Among RNs who received training, there was variability in their comfort when providing clinical care guided by the iCPR tools. This was expressed as resistance to the delegation portion of the intervention regarding adherence to the iCPRs, staying within their scope of practice, and compatibility with their clinical skills.

Some nurses felt organizational resistance to operationalizing the delegation protocol, which required nurses to practice to the full extent of their qualifications. One nurse leader expressed that the intervention enables nurses to function within the bounds of what they are licensed to do, but existing clinic workflows may not be able to accommodate them.

*It’s asking our nurses to work at the top of our license. The problem is that it takes—the way that they've worked in urgent care for so many years, it causes a change in that workflow…Some of the leaders have been more willing to facilitate that process, and some have not.*

[L 2.14]

Nurses expressed discomfort and uncertainty when triaging patients with cough-related symptoms, particularly when required to perform comprehensive respiratory assessments such as auscultating lung sounds. This hesitation often stemmed from fear of missing critical findings and potentially causing harm, which made them reluctant to fully engage with the iCPRs designed for these cases.

*…there’s a lot of nurses who do not like the cough part. They don't like to listen to lungs, and the reason is they're afraid they're going to miss something and harm patients.*

[L 3.20]

Educating nurses was highlighted as a potential means of addressing these barriers. One nurse leader shared:

*Training of new people training is huge, so I think and many people have to be hands on… mixture of hands-on CBT [Computer Based Training] all of that to reach the nurses…but I think training is key.*

[L 3.20]

Conversely, nurses reported a strong sense of satisfaction with the sore throat iCPR, noting its simplicity and efficiency, allowing patient assessments to be completed in as little as 15 minutes.

*The sore throat one we'll use constantly. That is very much something that has been, in my opinion, and I think our providers, everybody really, has been very successful.*

[N 3.3]

### Influence of Openness to Change and a Nurse-Supportive Clinic Culture

#### Nurse Factors

A supportive clinic culture, openness to change, and positivity toward nurse support influenced the use and sustainability of the iCPR-guided nurse delegation workflow. Within clinic culture, the perceptions of support from providers and patients emerged as subthemes, and leadership support was seen as crucial in facilitating the usage of the iCPR tool. Participants reported that leaders who actively listen to nurses and maintain ongoing dialogue foster a supportive environment that encourages new practices. Nurse leaders agreed that supporting staff was a success factor.

*I think just making sure that staff are feeling confident and comfortable with the process, hearing where there’s issues…I think just hearing what the staff is experiencing, trying to support them through any issues that are coming up, and making sure that they’re confident in their training to conduct the visits.*

[L 3.11]

*Ongoing discussions with the nursing team to make sure that they feel supported*.

[L 3.1]

The overall clinic culture was perceived to be particularly critical. One nurse described the culture of being open to novel processes.

*…I think that our culture, to use that term for sure, is exceptional in that way. As far as being open to adopting things. Moving forward with nursing skills and nursing involvement in clinic practice is certainly something we focus on as a group and with our leadership.*

[N 3.1]

Another participant reported the benefit of a shared value system within the clinic:

*We have an excellent connection between each other. I think we all work for each other and with each other. There is a shared value system there that was very apparent and that is healthy in many ways… If my boss wasn't that interested in this, I think that we as a group would still be very interested together. We would have just done it by ourselves.*

[N 3.1]

Moreover, nurses expressed aspects of professional fulfillment due to spending more time with patients:

*Talking to the patients is great. Sometimes it’s nice, the ones that you talk to, then you get to do a nurse visit*.

[N 1.7]

*Once again, I think it’s great for us. It’s great for the patient. I think being accessible and giving our patients what they need is—just been a really nice experience.*

[N 3.2]

#### Provider Factors

Factors related to providers were found to be an essential subtheme of the clinic culture. Overall, the provider’s acceptability of the processes was viewed as necessary for the sustainability of the iCPR3 tool.

*We'd have to get the… medical director on board to really get the providers engaged with this. I do feel like providers are one of the bigger barriers. The nursing teams can do this work, but unless we work in collaboration, it'd be pretty hard to have it be sustainable.*

[L 2.14]

When medical directors showed interest in teaching and experimenting with new workflows that incurred time savings, the tool’s adoption by RNs was supported.

*If they perceive, or if it’s a reality that this program takes visits away from them, rather than gives them more time to see other patients. Then that could be a deterrent and also just their own understanding of how nursing practice can be used. I think in ambulatory it’s sometimes disinterested.*

[L 1.13]

*When the doctors like to teach…then it’s very helpful. Some doctors… don't even want to do the study…but the ones that want to teach then, that’s very helpful.*

[N 1.7]

Engagement from a medical director through collaboration was deemed important for facilitating clinic change.

*It’s doable, it’s just, again, it’s change management. It’s really having everybody on board. Probably something that would help it move along better would be all the leaders and medical director meeting and deciding this is the right process….*

[L 2.14]

#### Patient Factors

Patient engagement varied by location and was viewed as tightly connected to the clinic culture. Some nurses reported challenges in communicating the iCPR outcome to patients, especially when the patient had certain expectations connected to receiving antibiotics as part of the visit.

*We have a low compliance rate in our clinic. When they come to see us, sometimes even when you’re like, “You’re not gonna see a doctor. You’re just gonna see a nurse.” They just want to come in and get antibiotics… When they come in and see us, I’m really like, “You’re not gonna [get antibiotics],” but sometimes does not go over well with our patient population.*

[N 3.10]

A number of issues also described a conflict between the delegation process and the patient’s preference for provider type.

*We do get some that are not interested in having the nurse visit type because they are looking to discuss other remedies or other treatment options, or their complexity medically is sometimes a barrier.*

[N 3.18]

*I think our patient population is different than other clinics. I think that that has made it a little bit harder… We do get pushback from our patients. They’re like, ‘Well, you’re not a doctor,’…*

[N 3.10]

However, some patients appreciated the extra access to appointments and were willing to have a nurse visit and were satisfied with the nurse visit.

*I feel like patients are very agreeable and willing to come in to see a nurse, after we've explained exactly what’s gonna happen… Patients have been really appreciative. No cost, they get in the same day, which is really what they're looking for…*

[N 3.2]

#### Clinic Communication

General clinic communication was the final subtheme related to clinic culture. A nurse leader expressed interest in having more usage data about the study’s progress to communicate with the staff for positive reinforcement.

*…if people know the why we're doing it that helps…It really comes down to great patient care, antibiotic stewardship, how many visits we did. All of that. I think if we have that data, then as we train, we can bring that forward and show people why we're doing it.*

[L 3.20]

Nurse participants also reported satisfaction connected to the receipt of feedback on the iCPR use.

*I think it’s been very positive and we—you guys send out the statistics and it reinforces that we are doing a good job and that always obviously makes me feel good that you are making a difference and… because obviously it’s helping everybody. It’s helping our patients. It’s helping us*.

[N 3.2]

Group communication and decision-making as a team were noted as key to the sustainability of clinic initiatives.

*Again, it’s about everyone deciding together that this is the right path and the way we want to go. Then moving it forward as a group so that it’s not over here at this clinic but not at this clinic…It’s just making sure you've got both nursing and operations, if we're going to really operationalize something and put it into permanent status.*

[L 2.14]

Another participant emphasized the importance of teamwork among colleagues, noting the ability to share experiences and seek advice from others to navigate challenges.

*My co-workers, my colleagues, us working together was another really important piece… Have you done this? Did you run into that?*

[N 3.3]

In addition, communication tools were reported to provide support beyond programmatic issues and were seen as helpful in solving acute technology issues.

*The Webex group was helpful troubleshooting technology issues. They were really good with the patient care part, but, if a technology issue came up, the Webex group was really helpful.*

[N 3.6]

### Ongoing Need for Training and Support

Participants felt that additional training, specifically hands-on training, would enhance the program’s sustainability.

*… Can we retrain? Can we have champions at the site who can help? Training of new people training is huge, so I think and many people have to be hands-on. Is it a mixture of hands-on CBT [computer-based training]… I think training is key.*

[N 3.2]

Another participant reported that recurrent training would help with maintaining skills:

*Offering skills' refreshers every once in a while if this is gonna be an ongoing thing would be helpful too.*

[N 3.15]

*I think education and I think not just a one and done education…at the time of hire, at the time of roll out or sustainability, but then I almost think something yearly just for people to review, ask questions, do hands on skills again*

[L 3.20]

In the context of simulation training, a playground or safe, interactive environment was described to bridge this gap, providing a way for learners to practice skills without real-world consequences. One nurse leader was enthusiastic about ways to integrate hands-on training with the iCPR intervention.

*I would say the hands-on training was fantastic for the nurses. They really appreciated it. It made them feel much more comfortable… in a setting where they can use the playground and practice the swabbing and stuff… making sure we can do a real-world, full-picture visit for practice would be really helpful.*

[L 3.6]

Both nurse and nurse leader participants considered nursing leadership support to be part of the sustainability of this program. One of the nurse leaders described their approach as:

*Just making sure the nurses are comfortable and have the training and education that they need. If the cough part is a barrier, working to get them hands-on training, making sure they've got swabs that they need, stethoscopes that they need, and just being support for them. If they don't have a buddy in the clinic that day, maybe I step in and do it with them.*

[L 3.6]

### Recommendations Based on Participant Feedback

Participants provided several recommendations to enhance the sustainability of the iCPR tool in nursing practice (Table 1).

**Table 1.** Recommendations.

Recommendation	Representative quote
Identify clinic champions	“have champions at the site.” “we’re going to need a provider champion.”
Educate patients	Educate the patients more on “This is what we’re doing.”
Organize frequent lunch and learns	“circling back to another … skill driven practice type session might be helpful just to make sure … we still feel confident that we can conduct it and not feel too flustered.”
Perform triage in advance	“When the first person that gets the [triage] call says, “Oh, they have a cough or a sore throat.” That’s very helpful because right away it’s identified …"
Map physical workflows	“… in the future … you can see the layout of how things are, then you can maybe give a suggestion or tell us something to make a different flow that works somewhere else.”
Incorporate into routine practice and policy	“… has to make it a delegation process … more than anything.”

## Discussion

### Principal Findings

The examination of the perceptions of nurses and nurse leaders familiar with the implementation of a CDS-guided nurse intervention for ARIs revealed several barriers and facilitators to the sustainability of the tools’ use beyond the conclusion of the study. Factors perceived as contributing to sustainability emerged within 5 themes, including (1) the importance of staffing stability and capacity; (2) the impact of dedicated clinic resource availability; (3) variable nurse readiness with CDS-guided clinical care; (4) the influence of openness to change and a nurse-supportive clinic culture; and (5) the ongoing need for training and support. By touching on the characteristics of the intervention, clinic setting, and individuals, these themes highlight several CFIR domain constructs and strategies at the individual and organizational levels to be considered when seeking to develop a sustainable CDS-guided intervention [15,19]. The most pertinent constructs identified in this research are local attitudes and conditions, partnerships and connections, structural characteristics such as physical and work infrastructure, organizational culture, available resources and space, the roles of innovation deliverers (nurses) and recipients (patients), as well as strategies for tailoring and adapting the use of the iCPR tool.

### Individual-Level Strategies

Our analysis highlighted that nurses expressed lower confidence when using the iCPR tool for conditions requiring more complex or subjective clinical assessments, such as listening to breath sounds, compared to workflows perceived as more straightforward, such as visual inspections for sore throat evaluations. Indeed, several participants indicated a preference for sustaining use of the sore throat iCPR workflow only. This gap in confidence suggests a need for targeted skill development. Simulation-based training offers a valuable strategy to build these skills in a realistic, low-risk environment. Regular simulations can help nurses practice nuanced assessments, receive real-time feedback, and strengthen clinical judgment. Incorporating iCPR-related scenarios into onboarding and ongoing education can reinforce competence and confidence. Fostering a culture of continuous learning, with structured supervision and feedback, is essential for supporting effective and sustained implementation [20]. In alignment with CFIR individual constructs [15], the identification of subject matter experts could further assist and support access to information and knowledge.

### Organizational-Level Strategies

As seen with many health care interventions, staffing levels were perceived as a key factor influencing long-term sustainability [21]. Inadequate ambulatory clinic nurse staffing impedes staff mastery of new procedures, restricts training time, and hinders comprehension of essential tools, such as EHR CDS modules [22]. Moreover, the increased complexity of ambulatory nursing requires nurse leaders to proactively adjust outpatient staffing models to meet evolving demands [23]. Staffing models were not adjusted to accommodate iCPR3; instead, participating units were only required to have at least one nurse. This model worked well for some clinics but not all. In settings with limited staffing, nurses may be forced to prioritize urgent clinical tasks over engaging with new initiatives, making it difficult to implement interventions effectively. Our findings suggest that a “one size fits all” approach may be inadequate. Instead, staffing models should be tailored to the needs of each clinic, particularly when new interventions are introduced. Due to national nursing shortages, calibrating nurse staffing is more important than ever before [24]. Adequate staffing enables nurses to focus on their patient care activities [24,25], allowing for better adherence to the CPRs. Study participants highlighted encountering challenges integrating new hires, resulting from turnover, and upskilling nurses who have transferred from other specialty areas. Prior to intervention implementation, analyses of turnover rates, percentage of new hires, and overall patient volume should be considered to ensure appropriate staffing availability, aligning with the CFIR construct of general staffing levels to support the functional performance of the inner setting [16]. Strategic planning should incorporate anticipated staffing fluctuations and allow flexibility in response to sudden staffing interruptions. The use of alternative staffing models, such as virtual nursing resources, needs to be considered. While an efficient use of nursing resources in some settings [26], hybrid work environments may contribute to barriers to care coordination and in-person visits.

Availability of clinic resources, especially physical space, was emphasized as a barrier more than expected. Success with the iCPR workflow hinged on ready access to supplies, designated rooms for nurse consultations, a supportive physical layout, and timely provider availability. Compared to inpatient hospital care, the outpatient environment is known to often lack infrastructure support [27]. When nurses had to search for appropriate locations to assess patients upon arrival, both efficiency and patient experience suffered. In contrast, clinics that reserved rooms specifically for nurse visits reported smoother workflows and better outcomes. As per the CFIR physical infrastructure construct, the impact of the layout and configuration of the space affects functional performance [15]. In nonambulatory clinics, similar issues have been previously observed, indicating that both ambulatory and nonambulatory settings face challenges related to resources. Despite progress in CDS tools, considerable barriers remain, especially regarding workflow integration and resource allocation [27]. Future implementations should therefore include a thorough assessment of space and workflow needs, ensuring that dedicated nurse-visit rooms and adaptable layouts are built into the design from the onset [28].

Clinics with positive experiences using the iCPR tools often reported a strong organizational culture characterized by teamwork, openness to change, and leadership that valued nurse autonomy, clinical expertise, and collaborative practice. Supportive leadership and a team-based approach further enabled the effective coordination between nurses and providers required to implement and sustain a new CDS intervention. As recognized by updated CFIR constructs, local attitudes and beliefs influence future use [15]. Participants also emphasized the importance of involving patients in shared decision-making, underscoring the need to respect patient preferences and set clear expectations during care encounters. This finding suggests that fostering a culture that empowers nurses, supports interdisciplinary collaboration, and centers patient engagement may be key elements to the successful nurse adoption of CDS tools in the ambulatory care setting [29].

Recognizing that the iCPR RCT took place in ambulatory settings connected to academic medical centers, there are insights that may translate to nonacademic centers. While community centers may be less resourced for experimentation and have underdeveloped nursing platforms, nurses in nonacademic settings often work at the top of their license out of necessity for patient throughput [30]. Prior to implementing the recommended strategies that connect to our findings, it will be important to compare workload patterns and assess the need for delegation protocols prior to implementing a similar intervention.

### Limitations

Several limitations should be noted. This study was conducted within academic medical centers, which are typically characterized by greater infrastructural resources and institutional support, potentially limiting the generalizability of findings for community or resource-constrained settings. Additionally, we did not account for the differences in institutional policies, patient populations, or resource constraints as part of our examination. While thematic saturation was achieved, the final sample size was relatively small, and the sample skewed toward nurses at one site and toward nurse leaders rather than frontline nursing staff, which may have introduced hierarchical bias in perceptions of implementation. However, given low intervention engagement during the RCT at the Utah and NYU sites and that Wisconsin had the most nurses using the tool, we believe the sample was a representative of the iCPR RCT site variability. Participant selection may have been subject to response bias, as we used a convenience sample and did not stratify responses based on participants’ familiarity with technology, experience, or frequency of tool usage. Because use of the intervention tools was part of the inclusion criteria, we did not assess the perspectives of nurses who did not perform a nurse visit or who did not participate in the RCT. In addition, participants were recruited and interviewed by known study personnel at their corresponding sites, which may have led to social desirability bias and impacted candor in participant responses. Lastly, this analysis does not include an examination of tool utilization or patient-level outcomes and their influence on perceived sustainability.

### Conclusion

This qualitative analysis underscores the multifaceted nature of sustaining a CDS-guided nurse iCPR intervention for ARIs in ambulatory care settings after a formal study period. The findings reveal that sustainability hinges not only on the design of the intervention but also on contextual factors such as staffing adequacy, resource availability, nurse confidence, organizational culture, and ongoing training. Importantly, variability in nurse comfort with CDS tools and differential success across clinic settings suggest that tailored implementation strategies are essential. Future efforts should prioritize clinic-specific readiness assessments, targeted skill development, and leadership engagement to enhance the long-term viability of nurse-led CDS interventions in clinical workflows.

## Supplementary material

10.2196/83567 Multimedia Appendix 1 iCPR survey tool questions.

## Abbreviations

ARI: acute respiratory infection
CDS: clinical decision support
CFIR: Consolidated Framework for Implementation Research
CPR: clinical prediction rules
EHR: electronic health record
iCPR: integrated clinical prediction rules
RCT: randomized controlled trial
RN: Registered Nurse
