# Supplementary material for: Barriers and Enablers for Sustaining Nurse-Led Use of Clinical Decision Support Tools for Antibiotic Stewardship: Qualitative Study
Source: JMIR Nurs. 2026 Mar 10;9:e83567. doi: 10.2196/83567 (PMC12974925; doi:10.2196/83567)
Supplement: Multimedia Appendix 1 [file nursing-v9-e83567-s001.docx]

**APPENDIX/SUPPLEMENTARY MATERIALS**

| **Table S1. iCPR Tool questions - RNs** |
| --- |
| Question |
| 1.     Could you please tell me a bit about your experience participating in the [insert how you typically refer to iCPR3] project? |
| 2.     What has helped support you in conducting nurse visits and using the [insert how you typically refer to iCPR3] tools? |
| 3.     In terms of support from the research study team, what was helpful for your participation in the [insert how you typically refer to iCPR3] project? |
| 4.     Can you think of any additional support or training the research study team could have offered that would have been helpful in preparing you to participate in [insert how you typically refer to iCPR3]? |
| 5.     What characteristics of your clinic do you think impact [insert how you typically refer to iCPR3] implementation the most (positively and negatively)? |
| 6.     Would you like to use the [insert how you typically refer to iCPR3] workflow after the study ends? Why or why not? |
| 7.     What support is needed to continue [insert how you typically refer to iCPR3] project beyond the end of the research study and ensure its success in the long-term? |
| 8.     Is there anything else you would like to share? |

| **Table S2. iCPR Tool questions - Leaders** |
| --- |
| Question |
| 1.     What do you see as the top 3 clinic or system barriers to the implementation of the [insert how you typically refer to iCPR3] (for nurses in your clinic/ for clinics at [your institution])? |
| 2.     Who do you think would be able to address these clinic or system barriers? Which ones? |
| 3.     What do you see as the top 3 clinic or systems facilitators to the implementation of the [insert how you typically refer to iCPR3] (for nurses in your clinic/ for clinics at [your institution])? |
| 4.     How interested is the organizational leadership in this [insert how you typically refer to iCPR3] implementation? What about physician leadership? |
| 5.     How ready is the clinical practice to permanently implement the [insert how you typically refer to iCPR3]? |
| 6.     What role could you have in ensuring successful implementation of [insert how you typically refer to iCPR3] (in your clinic/ at [your institution])? |
| 7.     Is there anything else you would like to share? |
